# China’s city-level carbon emissions during 1992–2017 based on the inter-calibration of nighttime light data

**DOI:** 10.1038/s41598-021-81754-y

**Published:** 2021-02-08

**Authors:** Jiandong Chen, Ming Gao, Shulei Cheng, Xin Liu, Wenxuan Hou, Malin Song, Ding Li, Wei Fan

**Affiliations:** 1 School of Public Administration, Southwestern University of Finance and Economics, Chengdu, China; 2 Curtin University Sustainability Policy Institute, Curtin University, Perth, Australia; 3 School of Finance, Shanghai Lixin University of Accounting and Finance, Shanghai, China; 4 University of Edinburgh Business School, University of Edinburgh, 29 Buccleuch Place, Edinburgh, UK; 5 School of Statistics and Applied Mathematics, Anhui University of Finance and Economics, Bengbu, China; 6 Institute of Development Studies, Southwestern University of Finance and Economics, Chengdu, China; 7 West Center for Economic Research, Southwestern University of Finance and Economics, Chengdu, China

**Keywords:** Energy and society, Environmental economics, Socioeconomic scenarios

## Abstract

Accurate, long-term, full-coverage carbon dioxide (CO_2_) data in units of prefecture-level cities are necessary for evaluations of CO_2_ emission reductions in China, which has become one of the world’s largest carbon-emitting countries. This study develops a novel method to match satellite-based Defense Meteorological Satellite Program’s Operational Landscan System (DMSP/OLS) and Suomi National Polar-orbiting Partnership’s Visible Infrared Imaging Radiometer Suite (NPP/VIIRS) nighttime light data, and estimates the CO_2_ emissions of 334 prefecture-level cities in China from 1992 to 2017. Results indicated that the eastern and coastal regions had higher carbon emissions, but their carbon intensity decreased more rapidly than other regions. Compared to previous studies, we provide the most extensive and long-term CO_2_ dataset to date, and these data will be of great value for further socioeconomic research. Specifically, this dataset provides a foundational data source for China’s future CO_2_ research and emission reduction strategies. Additionally, the methodology can be applied to other regions around the world.

## Introduction

China has experienced an acceleration of urbanization and industrialization and is now one of the largest emitters of carbon dioxide (CO_2_) globally^1^. Hence, an increasing number of scholars are focusing their efforts on the development of effective carbon emission reduction strategies and ways to promote a sustainable low-carbon economy^2,3^. However, these studies mainly have been concentrated at the national and provincial levels, and research on city-level carbon emissions remains scarce. One reason for this trend is that there exists a lack of officially published city-level data. Additionally, even though some cities have published detailed information on energy use, cross-sectional and consecutive year data are difficult to compare and the quality of the corresponding data is often very limited^4–6^. In reality, cities are the main carbon emitters globally, and constitute a very significant branch of the Chinese government because these places are in an intermediate position in the overall policy formulation and implementation framework for carbon emission mitigation efforts^7–10^. If only the national or provincial carbon emissions and corresponding driving forces receive attention, the heterogeneous characteristics of city-level carbon emissions will be ignored, and this will not be beneficial for the development of strategies that can support city-level sustainable development.

In light of these issues, some scholars have attempted to calculate China’s city-level carbon emissions. At present, the relevant literature can be classified into two categories on the basis of the different methods that were used. The first group directly estimates carbon emissions of several cities in certain years based on energy inventory data, which were collected from China City Statistical Yearbooks, Local Government Work Reports, or other relevant statistical sources in local governments^8,10–15^. In total, these studies collectively represent an extensive effort to organize relevant data and produce relatively reliable data on city-level carbon emissions. However, this category of research has several limitations: first, the city-level carbon emissions obtained to date cannot have a significantly wide spatial and temporal coverage; second, the energy inventory data collected on different cities may be of different scales, and may make influences on the comparability of the cities’ carbon emissions; and third, because the different studies adopted different study areas, periods, and sectors, there may be large discrepancies among the results, which is not helpful for further research on city-level carbon emissions.

The second group of studies mainly adopts nighttime light data, especially DMSP/OLS (Defense Meteorological Satellite Program’s Operational Landscan System) images, as a proxy tool to estimate carbon emissions^6,16–18^, and this approach has been widely accepted in a variety of research fields^19–22^. However, although DMSP/OLS data tend to fit well when used to measure carbon emissions, the research period cannot exceed 2013 because DMSP/OLS images are not available after 2013. Furthermore, even when another type of nighttime light data, such as NPP/VIIRS (Suomi National Polar-orbiting Partnership’s Visible Infrared Imaging Radiometer Suite) images, is used to generate data from 2012 onwards, there are evident discrepancies between the two types of data.

As time advanced, studying only the period of 1992–2013 was proven to be insufficient. Hence, a few studies have tried to extend the research period and estimate China’s city-level carbon emissions after 2013^23,24^. However, important limitations have been encountered, such as white noise in the NPP/VIIRS images, poor fitting models, and saturation that can lead to errors. In conclusion, although studies have found that nighttime light data can be used as a good proxy tool for estimating carbon emissions, few studies have successfully been able to provide accurate, long-term, full-coverage CO_2_ data in units of China’s prefecture-level cities because of the unavailability of corrected nighttime light data and inaccurate estimation methods.

To address the gaps in existing data on city-level carbon emissions in China, both Chen et al.^25^ and we have proposed an improved method for inter-calibrating the two sets of data, which can be used to obtain stable, long-term nighttime light data series that can facilitate carbon emission assessments and the development of effective mitigation strategies. Compared to Chen et al.^25^ and other studies, the contributions of this study are as follows: (1) we developed an accurate, easy-to-understand, and improved method for inter-calibration of DMSP/OLS and NPP/VIIRS datasets to estimate the nighttime lighting of 334 prefecture-level cities in China from 1992 to 2017. Compared to previous studies, we extended the research time span. These data can be used for research in various fields such as in estimating the population distribution, gross domestic productivity, and income per capita; (2) we adopted variant coefficient models and the normalized difference index (NDI) to quantify China’s city carbon emissions based on nighttime light data of cities. Compared to the PSO-BP (Particle Swarm Optimization-Back Propagation) model adopted by Chen et al.^25^, our models improved the data fitting and provided a reliable data source for further studies; and (3) we explored the drivers of cities’ carbon emission increment in China, and provided useful reference for effective carbon emission reduction policies.

## Methods

### Study area and materials

As there are regional differences in China, and provincial carbon emissions data for Tibet, Hong Kong, Macau, and Taiwan were unavailable, our research scope excluded these regions. To match existing official statistics and reflect the continuous changes in China’s carbon emissions, our research period spanned from 1992 to 2017.

In this study, DMSP/OLS and NPP/VIIRS nighttime light imagery were adopted to simulate China’s city-level carbon emissions. The DMSP/OLS nighttime light imagery was primarily obtained from the F10, F12, F14, F16, and F18 satellite sensors in use during 1992–2013; a total of 34 years of synthetic images were available, and light noise caused by fires and other incidental background noise were removed. The product showed annual average composites of stable and persistent NTL with 30 arc-second grids (~ 1 km). However, the limitation of the product was the six-bit data quantification, which leads to over saturation, particularly in large cities. The NPP/VIIRS nighttime light imagery were derived from a polar orbiting Earth observation satellite during 2012–2020, and these data were more effective in discriminating various light sources than the DMSP/OLS data, which helped to prevent the oversaturation of bright parts in the areas of light^26^. Compared to the DMSP/OLS stable NTL product, the NPP/VIIRS product improved the spatial resolution to 15 arc-second grids (~ 500 m) and the data quantification to 14-bit.

### Data preprocessing

For the DMSP/OLS images, by considering that the size of each grid cell decreased with increasing latitude, we projected the synthetic images of 34 years as Lambert equal area projections and resampled them at a spatial resolution of 1 km to reduce the impacts of grid cell changes. For the NPP/VIIRS images, we averaged the monthly images to obtain annual images and then used the same method as that for the DMSP/OLS data to resample and obtain nighttime light datasets with a consistent spatial resolution. Additionally, although the NPP/VIIRS images filtered out the effects of stray lights, moonlight, and cloud coverage, they retained noise from auroras, fires, boats, and other temporary lights. Therefore, some scholars have used the bright areas of DMSP/OLS imagery in 2013 as a mask to extract NPP/VIIRS images and remove the influence of white noise^27^. However, this method ignores the new bright areas during 2014–2017. Therefore, we adopted 0.3 nW m^−2^ sr^−1^ as the threshold to remove the noise, which is consistent with previous studies^28,29^.

### DMSP/OLS nighttime light image data correction

To strengthen the continuity and comparability of the nighttime light data from DMSP/OLS, we adopted the invariant region method, described by Wu et al.^30^, to inter-calibrate DMSP/OLS images; this was done to improve the accuracy of the data after inter-calibration. Additionally, we selected Hegang City, Heilongjiang Province, as the constant target area, which is consistent with previous studies^31–33^. There are two kinds of images that can be used as reference in the inter-calibration of DMSP/OLS nighttime light images; one group consists of the images from satellite F16 in 2007^31,33^, and the other group is formed from the global Radiance Calibrated Nighttime Lights (RCNTL) data in 2006 as a reference^30,34^. For the first and second inter-calibration methods, we adopted a second-order regression function for each satellite and a power function because the results did not exceed the upper limit of NPP/VIIRS data, respectively, because these results had a higher R^2^ value in each case than that of other functions^30,33^. These results are presented in Appendix Tables A2 and A3, respectively.

Then, we adopted an intra-annual composition to utilize the data in the same year provided by the different satellite sensors and improve the stability of lit pixels (such as F14 in 2001 and F15 in 2001). The model is as follows:

1 $$DN_{{\left( {n,i} \right)}} = \left\{ {\begin{array}{*{20}l} 0 \hfill & {DN_{{\left( {n,i} \right)}}^{a} = 0\& DN_{{\left( {n,i} \right)}}^{b} = 0} \hfill \\ {\frac{{(DN_{{\left( {n,i} \right)}}^{a} + DN_{{\left( {n,i} \right)}}^{b} )}}{2}} \hfill & {otherwise} \hfill \\ \end{array} } \right.$$

where $DN_{{\left( {n,i} \right)}}$ represents the DN (digital number) values of the $i^{th}$ lit pixel from two kinds of satellite sensors in the $n^{th}$ year ($n = 1994,1997 - 2007$).

### The inter-calibration between DMSP/OLS and NPP/VIIRS

Because the DMSP/OLS and VIIRS nighttime light images are two different sets of data, the data cannot be matched directly. The discrepancies in the data primarily exist because the spatial resolutions of the two sets of data are different, the points of spread functions of the two sensors are different, and NPP/VIIRS has a stronger capacity to identify low light levels^29^.

Hence, it was necessary to establish a relationship between the DMSP/OLS and NPP/VIIRS nighttime light images, and achieve a uniform scale for the two datasets. Since both DMSP/OLS and NPP/VIIRS provided images in 2013, we extracted the city-level mean DN values in 2013 from the two datasets and explored their relationships. Moreover, we had two types of inter-calibrated DMSP/OLS images based on the proposed inter-calibration: the individually adopted images from the satellite F16 in 2007 and the global RCNTL reference image in 2006. To conduct the comparison, we adopted the two sets of DMSP/OLS data respectively, to fit the NPP/VIIRS data. For simplicity, we use $D_{1}$, $D_{2}$, and $V$ to represent the DMSP/OLS data adopted from satellite F16 in 2007, that adopted from RCNTL in 2006, and the NPP/VIIRS data, respectively.

Then, we adopted a power function to fit the relationship between the mean pixel value of DMSP/OLS and NPP/VIIRS, which is consistent with previous studies^29,35^. The models constructed are as follows:

2 $$D_{1} = \alpha V^{\beta }$$

3 $$D_{2} = \lambda V^{\theta }$$

where $\alpha$, $\beta$, $\lambda$, and $\theta$ represent equation parameters.

Additionally, in accordance with the characteristics and changing rules of the nighttime light imagery, the DN value of a pixel on the light image in the following year should not be less than that in the previous year. Based on this inference, corrections of the multi-year stable bright pixel images of DMSP/OLS and NPP/VIIRS were performed using Eq. (4).

4 $$DN_{{\left( {n,i} \right)}} = \left\{ \begin{gathered} DN_{{\left( {n - 1,i} \right)}}^{{}} \begin{array}{*{20}c} , & {DN_{{\left( {n - 1,i} \right)}}^{{}} > DN_{{\left( {n,i} \right)}}^{{}} } \\ \end{array} \hfill \\ DN_{{\left( {n,i} \right)}}^{{}} \begin{array}{*{20}c} , & {otherwise} \\ \end{array} \hfill \\ \end{gathered} \right.$$

### Use of nighttime light data as a proxy to estimate CO_2_ emissions

As China does not have an official source of carbon emissions data, we adopted a method provided by the Intergovernmental Panel on Climate Change to estimate carbon emissions from the national energy consumption data; this approach has been used in many studies^36–39^. The corresponding equation is as follows:

5 $$CO_{2}^{t} = \sum\limits_{i = 1}^{30} {CO_{2,i}^{t} } = \sum\limits_{i = 1}^{30} {\sum\limits_{j = 1}^{17} {\left[ {E_{ij}^{t} \times } \right.} } \left. {LCV_{ij}^{t} \times CC_{ij}^{t} \times COF_{ij}^{t} \times \frac{44}{{12}}} \right]$$

where $CO_{i,2}^{t}$ represents the provincial carbon emissions, million tons; $E_{ij}^{t}$ represents the $j^{th}$ type of energy use in province $i$; $LCV_{ij}^{t}$ is the low calorific value of the $j^{th}$ energy consumption; $CC_{ij}^{t}$ is the carbon content of the $j^{th}$ energy source; and $COF_{ij}^{t}$ is the carbon oxidation factor of the $j^{th}$ energy source.

In order to capture the differences between provinces, we also adopted a panel regression model to fit the relationship between the provincial sum of digital number (SDN) values and carbon emissions, which is an approach consistent with previous studies^6,23^. The econometric model was constructed as follows:

6 $$CO_{2,it}^{{}} = \omega SDN_{it}^{{}} + \gamma_{i} + \varepsilon_{it}$$

where $SDN_{it}^{{}}$ represents sum of digital number, $\omega_{it}^{{}}$ is the estimated coefficient and $\gamma_{it}^{{}}$ is the fixed-effect, which reflects the differences between provinces.

Additionally, the panel regression results between provincial carbon emissions and SDN values (see Table 1) show that although model (d) fitted the data well, the coefficients of time fixed effects during 1992–1996 were unavailable because of the lack of provincial carbon emissions data during 1992–1996. As model (c) had better fitting effects than model (b) but might produce negative values when obtaining estimates for cities with lower DN values, we adopted the NDI concept to combine models (b) and (c), thereby avoiding negative values and improving the continuity of the results. The model is as follows:

7 $$NDI = \frac{{\left| {\left. {CO_{2,it}^{t} \bmod {\text{el}}({\text{c}}) - CO_{2,it}^{t} \bmod {\text{el}}({\text{b}})} \right|} \right.}}{{\left. {CO_{2,it}^{t} \bmod {\text{el}}({\text{c}}) + CO_{2,it}^{t} \bmod {\text{el}}({\text{b}})} \right|}}$$

8 $$CO_{2,it}^{t} = \left\{ \begin{gathered} \begin{array}{*{20}c} {CO_{2,it}^{t} \bmod {\text{el}}({\text{c}})} & {,\begin{array}{*{20}c} {if} & {NDI < 0.1} \\ \end{array} } \\ \end{array} \hfill \\ \begin{array}{*{20}c} {CO_{2,it}^{t} \bmod {\text{el}}({\text{b}})} & {,otherwise} \\ \end{array} \hfill \\ \end{gathered} \right.$$

where $CO_{2,it}^{t} \bmod {\text{el}}({\text{c}})$ represents the city-level carbon emissions based on model (c) and $CO_{2,it}^{t} \bmod {\text{el}}({\text{b}})$ represents the city-level carbon emissions based on model (b). The threshold for the $NDI$ was set as 0.1, which is similar to Wu et al.^30^. If $NDI$ > 0.1, a significant gap between the two results is implied which might be due to the constant of model (c); therefore, we should adopt model (b) in such cases. Meanwhile, if $NDI$ < 0.1, this indicates that there is little gap, and model (c) should be adopted because it fitted the data well.

**Table 1:** Results for the panel regressions between the provincial carbon emissions and the SDN values.

Variables	Model (a)	Model (b)	Model (c)	Model (d)
	$C$	$C$	$C$	$C$
Coefficient	0.000502***	0.000300***	0.000300***	0.000250***
	(0.000026)	(0.000016)	(0.000017)	(0.000016)
N	630	630	630	630
R^2^	0.953	0.929	0.992	0.996
AIC	11.11	11.53	9.41	8.88

(1) ***Denote significance at the 1% level. (2) The values in parentheses are standard errors. (3) Models (a), (b), (c) and (d) represent the results estimated based on fixed-effect models, estimated by variant slope models, from the use of variant slopes and individual fixed effects, and from the use of variant slopes and individual and time fixed effects, respectively. (4) AIC represents the Akaike information criterion.

### Decomposition model of CO_2_ emissions

The combination of Kaya identity^40^ and Logarithmic Mean Divisia Index (LMDI) decomposition approach^41^ can be used to separate the contribution of drivers to CO_2_ emissions^42,43^. This approach is simple, not affected by the time-span, and can realize the zero residual decomposition^44,45^.

According to the Kaya identity, the IPAT equation of the CO_2_ emissions was built as follows:

9 $$\begin{gathered} C_{i} = \frac{{C_{i} }}{{GDP_{i} }} \times \frac{{GDP_{i} }}{{P_{i} }} \times P_{i} \\ \\ = EI_{i} \times EG_{i} \times P_{i} \\ \end{gathered}$$

where $C_{i}$, $GDP_{i}$, and $P_{i}$ represent the CO_2_ emissions, gross regional product, and population size in different cities, respectively; $i$ represents Chinese cities; $EI_{i} = {{C_{i} } \mathord{\left/ {\vphantom {{C_{i} } {GDP_{i} }}} \right. \kern-\nulldelimiterspace} {GDP_{i} }}$ and $EG_{i} = {{GDP_{i} } \mathord{\left/ {\vphantom {{GDP_{i} } {P_{i} }}} \right. \kern-\nulldelimiterspace} {P_{i} }}$ represent technical progress and economic growth of city $i$, respectively.

The LMDI decomposition approach was used to calculate the contribution of driving factors to the changes of CO_2_ emissions. We set the CO_2_ emissions for the reporting and base periods as $C_{i}^{t}$ and $C_{i}^{b}$, respectively. The change of CO_2_ emissions between the two periods could subsequently be decomposed into:

10 $$\Delta C_{i} = C_{i}^{t} - C_{i}^{b} = \Delta EI_{i} { + }\Delta EG_{i} { + }\Delta P_{i}$$

where $\Delta EI_{i}$, $\Delta EG_{i}$, and $\Delta P_{i}$ represent the contribution of technical progress, economic growth, and population size to the change of CO_2_ emissions, respectively, and the specific forms are as follows:

11 $$\Delta EI_{i} = L\left( {C_{i}^{t} ,C_{i}^{b} } \right) \times Ln\left( {\frac{{EI_{i}^{t} }}{{EI_{i}^{b} }}} \right)$$

12 $$\Delta ED_{i} = L\left( {C_{i}^{t} ,C_{i}^{b} } \right) \times Ln\left( {\frac{{EG_{i}^{t} }}{{EG_{i}^{b} }}} \right)$$

13 $$\Delta P_{i} = L\left( {C_{i}^{t} ,C_{i}^{b} } \right) \times Ln\left( {\frac{{P_{i}^{t} }}{{P_{i}^{b} }}} \right)$$

among these, $L\left( {C_{i}^{t} ,C_{i}^{b} } \right) = \frac{{C_{i}^{t} - C_{i}^{b} }}{{Ln(C_{i}^{t} ) - Ln(C_{i}^{b} )}}$, for $C_{i}^{t} \ne C_{i}^{b}$; $L\left( {C_{i}^{t} ,C_{i}^{b} } \right) = C_{i}^{t} = C_{i}^{b}$, for $C_{i}^{t} { = }C_{i}^{b}$.

## Results

### Estimation of nighttime light image in China

After applying the fitting relationships, we obtained $\alpha$, $\beta$, $\lambda$, and $\theta$ in Eqs. (2) and (3) as 5.7005, 0.7248, 6.7214, and 0.7197, respectively. The R^2^ values of the relationships of inter-calibrated NPP/VIIRS and $D_{1}$, $D_{2}$ were 0.90 and 0.91, respectively, which are relatively close to the 0.915 estimate by Li et al.^29^. Then, we were able to convert the NPP/VIIRS image scale into the DMSP/OLS image scale, similar to Ma et al.^24^. To further eliminate the unstable pixel DN values and the obvious over-glow effect of DMSP/OLS data, we assumed that the bright areas of the corrected and transformed NPP/VIIRS image in 2017 were stable, used the data to extract the DMSP/OLS images, and replaced the remaining lit pixels with values of zero, which is consistent with Liu et al.^33^.

**Figure 1:**
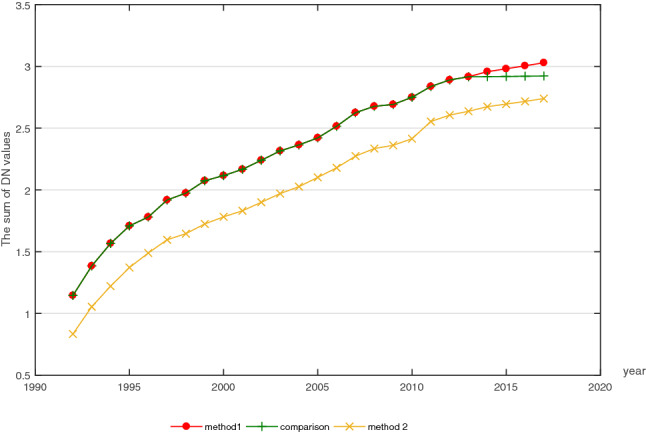
The total trend of the SDN values during 1992–2017 (unit of the SDN values: 10^7^). *Notes*: (1) method 1 represents the results based on $D_{1}$ data; (2) method 2 represents the results based on $D_{2}$ data; and (3) the comparison curve represents the results without the inter-calibration between DMSP/OLS and NPP/VIIRS images, revealing the gap between the two satellite’s images and indicating the significance of the inter-calibration.

Then, we estimated and obtained two sets of data on the city’s SDN values on the basis of two types of inter-calibrated DMSP/OLS images. The SDN values were estimated by two methods and the results are presented in Fig. 1. The comparison curve reveals the gap between the two satellite’s images and indicates the significance of the inter-calibration. Although the trends of the result estimated by method 1 and 2 are similar, the method 2 curve is higher than method 1, due to the problem of saturation. Therefore, we used the results of method 2.

Considering that there is a significant relationship between GDP statistics and nighttime light data^17,30,46,47^, we selected the provincial cross-sectional GDP statistics to perform linear regression with the nighttime light data in this study. The R^2^ values were > 0.8756 and the Akaike information criterion (AIC) values were small during 1992–2017, indicating that the calibrated nighttime light data could well characterize the GDP. Detailed results are presented in Appendix Table A4.

### Analysis of the city-level carbon emissions

Because of the large sample size of long-panel data, if individual points have different intercept terms, they may not fit the relationship between the two very well. Therefore, we further applied models that contained only the variable slope or both the variable slope and a constant, to perform the regressions. These results are presented in Table 1, and detailed information about the coefficients for the individual points, time, and variant slope are presented in Appendix Table A5.

As shown in Table 1, the slopes of all panel linear regression models were statistically significant. Additionally, the coefficient of the fixed-effect model was close to that estimated by Meng et al.^6^, thus indicating that our estimated linear relationship between carbon emissions and the SDN values was robust.

Moreover, because the R^2^ and AIC values can be used to evaluate the fitting results, all of the models fitted well. The R^2^ values were > 0.90, which indicated that over 90% of the changes in provincial carbon emissions in China could be explained by the SDN values; the AIC values were lower than the value (29.30) estimated by Meng et al.^6^. We observed that among the four models, model (d) fitted the data best, which brought the time fixed effects into consideration; this model explained over 99.6% of the changes in carbon emissions. Hence, we used model (d) to estimate the city-level carbon emissions during 1997–2017.

Moreover, as our data contained long-panel data of 26 years, the regression was likely affected by the possible presence of unit roots in the variables. Hence, the LLC (Levin, Lin, and Chu t test) and IPS (Im, Pesaran, and Shin Wald statistic test) panel unit root test results were presented for the series of provincial carbon emissions, as well as the SDN values and their first-order differences, which have been widely used in previous studies^42,48^. The results based on the LLC and IPS tests are presented in Table 2.

**Table 2:** Results for the panel unit root tests.

Variable	LLC		IPS	
	Level	First difference	Level	First difference
$C$	− 1.995**	− 6.219***	4.1241	− 9.2***
	(0.023)	(0.00)	(1.00)	(0.00)
SDN	− 6.0257***	− 7.886***	0.5888	− 7.7515***
	(0.00)	(0.00)	(0.722)	(0.00)

(1) Values in parentheses are the p-values. LLC denotes the Levin, Lin, and Chu t test; IPS denotes the Im, Pesaran, and Shin Wald statistic test. (2) The LLC and IPS tests for all of the series include an intercept term. (3) ** and *** denote the null hypotheses of a unit root at the 5% and 1% significance level, respectively. (4) SDN denotes the sum of digital number.

The results for both the LLC test and IPS test indicated that all variables appeared to be first-order single integer [I (1)] simultaneously. Hence, it was necessary to conduct panel cointegration tests and estimate the long-run equilibrium relationship among the variables. We adopted the Pedroni residual method^49^, which has been widely applied widely in various studies^50–52^. The results for the panel cointegration test statistics are presented in Table 3.

**Table 3:** Results for the panel cointegration tests based on the Pedroni residual method.

Test equation	Regression C SDN
Panel PP statistic	− 3.5974***
	(0.0002)
Panel ADF statistic	− 5.424***
	(0.00)
Group PP statistic	− 3.2135***
	(0.0007)
Group ADF statistic	− 5.9127***
	(0.00)

(1) ***Denote significance at the 1% level. (2) The values in parentheses are the p-values. (3) SDN denotes the sum of digital number.

From the statistical results, all the tests led us to significantly reject the null hypothesis of no cointegration, thereby suggesting a cointegration relationship among the provincial carbon emissions and SDN values. Hence, our econometric models for estimating carbon emissions were reliable and reasonable.

**Figure 2:**
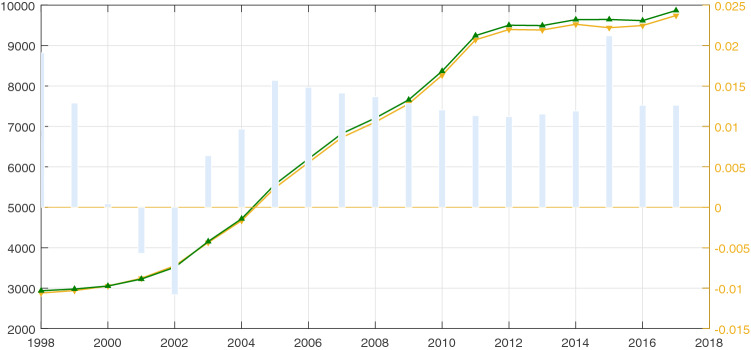
Comparison of our simulated total carbon emissions and actual total carbon emissions estimated from provincial energy balance tables during 1997–2017 (unit: million tons).

To test the robustness of the results obtained by our proposed method, we reported on the gaps between our estimated total emissions and published total emissions; these results are shown in Fig. 2. Simultaneously, we estimated the error rate between our simulated total carbon emissions and published total carbon emissions as “simulated carbon emissions / actual carbon emissions”, and these data are also presented in Fig. 2.

Visually, our simulated results were very close to the total values from the provincial energy balance tables. At the same time, it was evident that all of the error rates were far less than 0.05 million tons (the biggest absolute value of the error rate was around 2.11%). The following three key time nodes were of interest: 1997, 1999, and 2010. Evidently, the total carbon emissions increased during 1992–1997, while they decreased during 1997–1999. During 2000 to 2012, the carbon emissions increased rapidly, which is similar to the conclusions provided by Meng et al.^6^ and Su et al.^18^. However, from 2010 to 2017, the growth rates of total carbon emissions were relatively lower than before, which may have been a result of the strict limitations placed on emissions in policies proposed by the Chinese government^38,39,53^.

**Figure 3:**
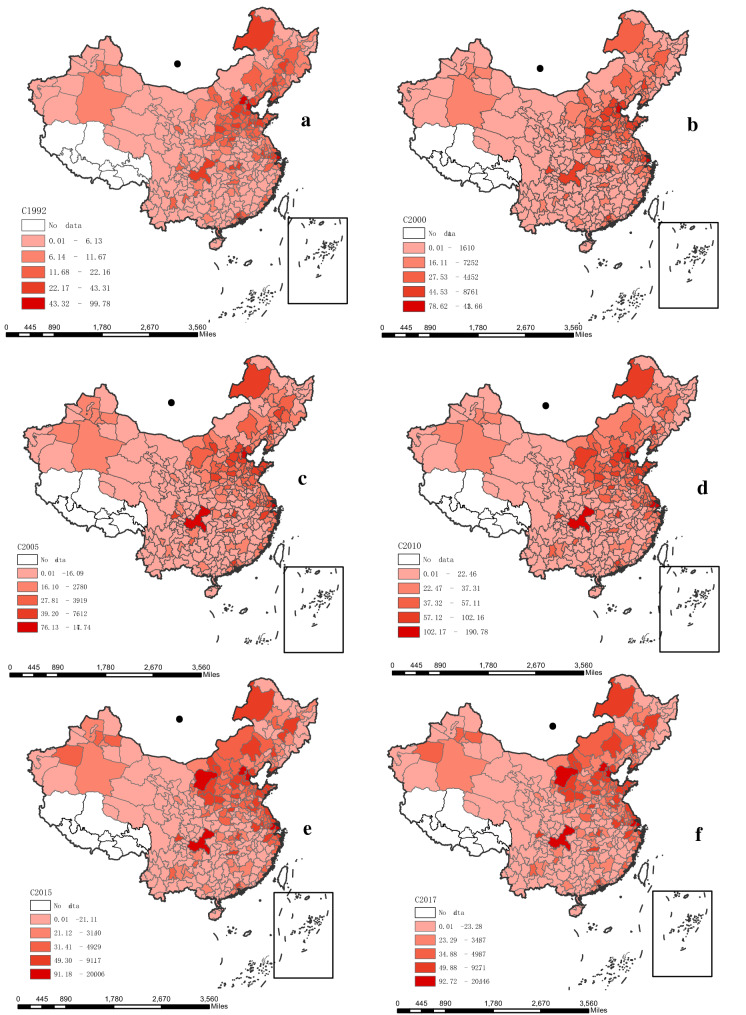
Spatial and temporal pattern of China’s city-level carbon emissions during 1992–2017 (unit: million tons). (**a**) 1992, (**b**) 2000, (**c**) 2005, (**d**) 2010, (**e**) 2015, and (**f**) 2017. *Note*: These images were made by ArcGIS 10.0. The version is available from: https://www.esri.com/en-us/arcgis/products/arcgis-maps-for-office/download.

In total, China’s national carbon emissions increased continuously from 1992 to 2017, during which the amount increased from 2239.58 to 9741.65 million tons with an average 6.23% annual growth rate. Additionally, the total growth rates for the periods of 1992–1996, 1997–1999, 2000–2012, and 2013–2017 were 40.58%, -0.11%, 207.93%, and 2.27%, respectively. As for the spatial and temporal variation trends of China’s city-level carbon emissions from 1992 to 2017, we constructed spatial maps that depict the results for 1992, 2000, 2005, 2010, 2015, and 2017, and these results are presented in Fig. 3; additional comprehensive figures are presented in Appendix Figs. 1–3.

### Comparison with previous studies

To further estimate the accuracy of the proposed method, we compared the estimated city-level carbon emissions with results from previous studies^13–15^. Specifically, we used the data from our proposed method and carried out regressions with their corresponding data; the results are presented in Table 4.

**Table 4:** Comparison of the results of our proposed method with those of Shan et al.^15^, Cai et al.^13^, and Jing et al.^14^.

Variables	Model (1)	Model (2)	Model (3)
	$C$	$C$	$C$
N	181	287	41
R^2^	0.8417	0.8464	0.9163

(1) The regression model is: $C = \alpha { + }\beta X{ + }\mu$, where $C$ represents the CO_2_ emissions of this study, $\mu$ is the residual term, and $X$ represents the CO_2_ emissions of Shan et al.^15^, Cai et al.^13^, and Jing et al.^14^, respectively. (2) Models (1), (2), and (3) represent the results compared with the data proposed by Shan et al.^15^, Cai et al.^13^, and Jing et al.^14^, respectively. (3) We focused on the similarity of CO_2_ emissions of this study with the other studies indicated here; thus, we have only provided the R^2^ value. (4) N is the number of cities.

In accordance with the coefficients of determination provided in Table 4, the results indicate that our corresponding data were 84.17% similar to 187 cities’ carbon emissions in 2010 as estimated by Shan et al.^15^, 84.64% similar to 287 cities’ carbon emissions in 2012 as estimated by Cai et al.^13^, and 91.63% similar to 41 cities’ carbon emissions in 2010 as estimated by Jing^14^. At the same time, because there were also some scholars who calculated carbon emission data for a few select cities in other years or over a certain period^11,12^, we also carried out comparisons with these data to test the accuracy of our results. Bi et al.^11^ measured Nanjing city’s carbon emissions from 2002 to 2009 based on six sectors in the energy inventory, and these data not only included emissions related to energy consumption, but also process- and waste-related emissions. Evidently, our corresponding estimated data were very consistent with those of Bi et al.^11^ as the R^2^ was almost 0.98. At the same time, our results were also consistent with the carbon emissions for several cities estimated by Mi et al.^12^, and the R^2^ was approximately 0.91.

Although our results were close to those of previous studies, there were also some differences. These differences may have stemmed from the different accounting methods used (the previous studies mainly relied on the city-level energy inventories from China City Statistical Yearbooks, Local Government Work Reports, and other relevant statistical sources of local governments), the errors caused by econometric models, or the errors from the estimated provincial carbon emissions.

In summary, because of the limited quality and availability of city-level energy inventories, we have proposed a new method for estimating China’ city-level carbon emissions based on the inter-calibration between DMSP/OLS and NPP/VIIRS data. Our results for 1992–2017 were very consistent with the results of previous studies. Thus, this technique should be useful for providing reliable city-level carbon emission data over a wide spatial scale in China, which will be beneficial for future academic and policy research.

### Analysis of carbon emission drivers

**Figure 4:**
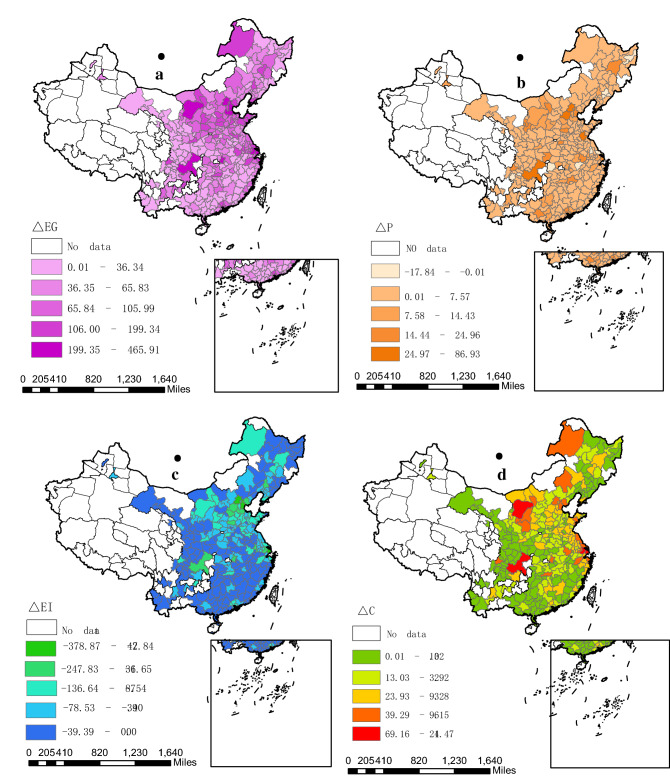
Spatial distribution of drivers of China’s city-level carbon emission increment during 1992–2017 (unit: million tons). (**a**) The contribution of economic growth to emission increment; (**b**) The contribution of population size to emission increment; (**c**) The contribution of technological progress to emission increment; (**d**) The emission increment of each city. *Notes*: (1) The gross domestic product (GDP) and population data of each city are from China City Statistical Yearbook, the statistical yearbook of each city, and the government bulletin. A few missing values have been supplemented by interpolation method. (2) Because of the lack of population and GDP data for some cities, 285 cities are covered here, excluding Hong Kong, Macao, and Taiwan. (3) These images were made by ArcGIS 10.0. The version is available from: https://www.esri.com/en-us/arcgis/products/arcgis-maps-for-office/download.

To further explore the changing trend of carbon emission increment in Chinese prefecture-level cities, we considered 1992 as the base period and 2017 as the report period, and combined with Kaya identity and LMDI decomposition approach, calculated the contribution of population size, economic growth, and technical progress to each city’s emission increment, respectively. Figure 4 shows the distribution of each driver increment. The results indicated that, in general, the CO_2_ emissions of 285 cities increased from 1992 to 2017, as shown in Fig. 4d. The primary reason is that industrialization and urbanization processes have been advancing rapidly due to reformation and expansion of China, and fossil-fuel energy consumption has been increasing annually in both production and residential sectors. Using the decomposition analysis of the driving factors of carbon emissions, we observed that, firstly, emission intensity was an indirect indicator of a city’s emission reduction technology, its contribution to the emission increment was negative, which implied that a city’s CO_2_ emissions were more likely to be reduced with an increase in the level of the city’s emission reduction technology. Secondly, the contribution of economic growth and population size to the emission increment was positive, which indicated that the city’s CO_2_ emissions were more likely to increase with the increase in the economic development and population density. However, the positive contribution of economic growth to the emission increment was greater than that of population growth.

From the perspective of cities, we observed that the cities with the highest emission increment can be broadly divided into two types: the first comprises the regional central cities dominated by Chongqing, Shanghai, Suzhou, Chengdu, Tianjin, Wuhan, and Beijing. Most of these cities are located in the eastern coastal areas and the central and western plains of China, where the population is dense and the degree of industrialization is extremely high. The second comprises the resource-based cities of Ordos, Hulunbeir, Yulin, Tangshan, Chifeng, etc., most of which are rich in coal, oil, and other mineral resources. For example, Ordos, whose annual raw coal production accounts for about one-sixth of the country’s raw coal production, is China’s largest coal producing city, and is commonly known as China’s “coal capital”. In addition, most of these two types of cities are dominated by iron and steel, coking, thermal power, cement, and casting industries, resulting in high fossil-fuel energy consumption and CO_2_ emissions.

From the perspective of driving factors, first, technological impact is the key factor for a city’s emission reduction. Among 285 prefecture-level cities in China, Shanghai, Tianjin, Beijing, Cangzhou, Suzhou, Wuhan, Chongqing, Shijiazhuang, Nanjing, and Guangzhou have the highest technological impact. These cities are primarily distributed in the eastern coastal areas and the central and western regional centers, as shown in Fig. 4c. The phenomenon is consistent with the actual layout of China’s regional development. These regions have gathered a large number of advantageous enterprises, and are the fastest and most advanced regions in China’s technological progress, especially in the application of low-carbon innovative technology^54^. Moreover, economically developed areas will also absorb more high-quality human capital than the rest. The participation of innovative talents has empowered the enterprises to implement carbon emission reduction technologies^55^. Secondly, economic and population growth are the primary factors for urban emission increase, as shown in Fig. 4a,b. Considering that China is in the critical stage of industrialization development, most of the industrial enterprises are expanding, large-scale input of factors is inevitable, and economic growth and human survival are inseparable from fossil-fuel energy consumption, resulting in high carbon dioxide emissions^56^.

In addition, the contribution of population size to emission increment in a few cities was negative, which may be because of the decrease of population in these cities in the reporting period compared to the base period. Specifically, firstly, some cities have poor development and are affected by the siphon effect of surrounding big cities, which leads to the loss of population every year. Additionally, the births in these cities are lower than deaths, which make the natural population growth rate negative for many years, such as Nantong city. Secondly, some urban administrative areas have changed, and the reduction of administrative areas will cause the decline of population size. For example, Suqian, which is under the jurisdiction of Yangzhou City, was upgraded to a prefecture-level city in 1996. Meishan and Hongya counties were established from Leshan City in 1997, and Zongyang County of Anqing city was under the jurisdiction of Tongling City in 2016.

## Discussion

Because cities have an intermediate position in the overall policy formulation and implementation framework for carbon emission mitigation efforts^8–10^, our data will be helpful for identifying the heterogeneous characteristics of city-level carbon emissions that can lead to further insights into the emission–economic nexus and the development of the most effective mitigation actions instead of only focusing only on national or provincial carbon emissions.

Based on the integration of two nighttime light datasets, we used the variant coefficient model and NDI index to estimate China’s city-level carbon emissions during 1992–2017. Then, to test the reliability of our data, we conducted comparisons with the data provided by other scholars. Given that detailed carbon emission data estimated by nighttime light data were not available and comparisons among different categories would be valuable, we adopted some relatively comprehensive emissions data calculated by energy inventories as a benchmark for the comparative analyses. The analyses showed that our results were 84.17–91.63% similar to previous city-level results reported for China^13–15^. These findings imply that our estimated prefecture-level carbon emissions data are reliable and valid.

Next, we individually estimated each city’s emission during 1992–2017. These results indicated that China’s high carbon emissions regions were clearly agglomerated in eastern coastal China, such as in the Beijing–Tianjin–Hebei region, Yangtze River Delta, and Pearl River Delta. At the same time, several cities in energy intensive provinces and mega-cities such as those in Inner Mongolia, Xinjiang, and Chongqing also had high carbon emissions. The counts of high-emission regions showed a trend of gradual spread.

To identify the causes of China’s CO_2_ emissions increase, we explored three aspects: technological progress, economic growth, and population size. From the results, we conclude that economic growth and population size are the boosters of a city’s emission growth, which is consistent with the conclusions drawn by Dong et al.^57^ based on global samples and Ding and Li^53^ based on China’s provincial samples. In contrast, this study is based on the city samples, and has a long time span; therefore, it can better capture the causes of city’s emission increase from the perspective of socioeconomic development. Moreover, Wang et al.^58^ and population control, but methods should be adopted to achieve a balance between socioeconomic development and carbon emission reduction. Some scholars believe that to achieve a balance, we may need to rely on improving energy efficiency and optimizing industrial structure, and the key to all these lies in technological progress^45,59^. Theoretically, the impact of technological progress on carbon emission reduction will first be reflected in the reduction of fossil-fuel energy demand. For example, using technological innovation, we can develop robust clean energy sources to replace fossil-fuel energy to meet the needs of economic development and human survival. This view is consistent with that of Brännlund et al.^60^. Simultaneously, in the power, transportation, construction, metallurgy, chemical, petrochemical and other industries with heavy pollution, we should strengthen technological innovation to promote the efficiency of carbon emission reduction. For example, clean and efficient use of coal resources, green development of oil–gas and coalbed methane, installations of carbon capture and storage equipment may be implemented.

In conclusion, this study adopted nighttime light data as a proxy tool to estimate China’s carbon emissions and obtain accurate, long-term, full-coverage CO_2_ data at the scale of prefecture-level cities, and the data was validated with corresponding data on carbon emissions calculated by conventional methods. On the basis of estimated city-level carbon emissions and factor decomposition analysis, we can identify the drivers and heterogeneity characteristics of city-level carbon emission increment, and provide an effective policy tool for the government to implement carbon emission reduction strategies. Notably, the dataset presented here will be useful for further studies that analyze the budget allocation of cities’ carbon emission rights, efficiency evaluations of city-level emission reductions, etc. Additionally, based on the proposed method for inter-calibrating the sets of DMSP/OLS and NPP/VIIRS data, we obtained continuous and stable nighttime light data during 1992–2017. As a basic data source, this nighttime light data is of great value and has broad application prospects in many research fields, particularly because it can be combined easily with other basic data such as data on the population size, economic activities, and energy use.

## Supplementary information

Supplementary Information.

## Data Availability

The data that support the findings of this study is available from the corresponding authors upon request.
