# Supplementary material for: China’s city-level carbon emissions during 1992–2017 based on the inter-calibration of nighttime light data
Source: Sci Rep. 2021 Feb 8;11:3323. doi: 10.1038/s41598-021-81754-y (PMC7870850; doi:10.1038/s41598-021-81754-y)
Supplement: Supplementary file 1 — Supplementary Information. [file 41598_2021_81754_MOESM1_ESM.docx]

Article Title:

China’s city-level carbon emissions during 1992–2017 based on the inter-calibration of nighttime light data

Author List

Jiandong Chen^a^ , Ming Gao^a^, Shulei Cheng^a^, Xin Liu^b^, Wenxuan Hou^c,d^, Malin Song^e^, Ding Li^f*^ , Wei Fan^g*^

^a^School of Public Administration, Southwestern University of Finance and Economics, Chengdu, China

^b^Curtin University Sustainability Policy Institute, Curtin University, Perth, Australia

^c^School of Finance, Shanghai Lixin University of Accounting and Finance, Shanghai, China

^d^University of Edinburgh Business School, University of Edinburgh, 29 Buccleuch Place, Edinburgh

^e^School of Statistics and Applied Mathematics, Anhui University of Finance and Economics, Bengbu, China

^f^Institute of Development Studies, Southwestern University of Finance and Economics, Chengdu, China

^g^West Center for Economic Research, Southwestern University of Finance and Economics, Chengdu, China

*Corresponding author: Ding Li, liding@vip.sina.com; Tel.: +86 13980676565.

Wei Fan, fw_mailbox@163.com; Tel.: +86 17381560809.

Appendix A

**Appendix Table A1.** The rank of city-level carbon emissions.

| The rank | Low (L) | Relatively low (RL) | Moderate (M) | Moderately high (MH) | High (H) |
| --- | --- | --- | --- | --- | --- |
| Dividing criteria | $(0,\bar{C}-s)$ | $(\bar{C}-s,\bar{C}+s)$ | $(\bar{C}+s,\bar{C}+1.5s)$ | $(\bar{C}+1.5s,\bar{C}+2.5s)$ | $(\bar{C}+2.5s,+\infty)$ |

Note: $\bar{C}$ is the mean city-level carbon intensity and $s$ is the standard deviation in Chinese Mainland.

**Appendix Table A2.** Coefficients of the second-order regression models for NSL data (satellite F16 in 2007 was selected as the reference image).

| Satellite | Year | $a$ | $b$ | $c$ | R^2^ |
| --- | --- | --- | --- | --- | --- |
| F10 | 1992 | -0.0033071 | 1.303979 | 0.5859372 | 0.8041 |
| F10 | 1993 | -0.00505 | 1.539204 | -0.68518 | 0.8488 |
| F10 | 1994 | -0.00809 | 1.604506 | -0.90777 | 0.8446 |
| F12 | 1994 | -0.00532 | 1.376458 | 0.730064 | 0.8019 |
| F12 | 1995 | -0.0045 | 1.326454 | 0.103591 | 0.8369 |
| F12 | 1996 | -0.00145 | 1.165291 | 1.531492 | 0.8308 |
| F12 | 1997 | -0.00562 | 1.448678 | -1.33737 | 0.8453 |
| F12 | 1998 | -0.0030853 | 1.221947 | -0.7520454 | 0.8726 |
| F12 | 1999 | -0.00446 | 1.322207 | -0.2974 | 0.8809 |
| F14 | 1997 | -0.01329 | 1.887614 | 0.00768 | 0.8562 |
| F14 | 1998 | -0.01097 | 1.752748 | -1.34707 | 0.8761 |
| F14 | 1999 | -0.01519 | 1.978259 | -1.50935 | 0.8969 |
| F14 | 2000 | -0.00764 | 1.49186 | -0.47777 | 0.9002 |
| F14 | 2001 | -0.00878 | 1.545653 | -1.20883 | 0.9539 |
| F14 | 2002 | -0.00462 | 1.207015 | 1.110137 | 0.8694 |
| F14 | 2003 | -0.00469 | 1.235021 | 0.53707 | 0.9568 |
| F15 | 2000 | -0.00401 | 1.222058 | 0.312183 | 0.8038 |
| F15 | 2001 | -0.00577 | 1.349919 | -0.6197 | 0.8468 |
| F15 | 2002 | -0.00389 | 1.233141 | -1.29059 | 0.9485 |
| F15 | 2003 | -0.00685 | 1.403376 | 0.593287 | 0.91 |
| F15 | 2004 | -0.0032 | 1.180613 | 0.321 | 0.9443 |
| F15 | 2005 | -0.0012894 | 1.058695 | 1.738491 | 0.8576 |
| F15 | 2006 | 0.000502 | 1.018413 | 0.85234 | 0.9663 |
| F15 | 2007 | -0.00314 | 1.230329 | 0.362825 | 0.9454 |
| F16 | 2004 | 0.00187 | 0.815426 | 1.155062 | 0.9047 |
| F16 | 2005 | -0.0023 | 1.142824 | 0.236669 | 0.9497 |
| F16 | 2006 | -0.00032 | 1.048318 | 0.15859 | 0.9636 |
| F16 | 2007 | 0 | 1 | 0 | 1 |
| F16 | 2008 | 0.004289 | 0.740674 | 0.948619 | 0.9659 |
| F16 | 2009 | 0.01017 | 0.307661 | 2.809703 | 0.9232 |
| F18 | 2010 | 0.013237 | -0.07146 | 5.829088 | 0.8038 |
| F18 | 2011 | 0.008931 | 0.302637 | 3.511007 | 0.8221 |
| F18 | 2012 | 0.013104 | 0.080266 | 4.259585 | 0.9192 |
| F18 | 2013 | 0.011049 | 0.079695 | 2.589046 | 0.8411 |

Note: $a$, $b$, and $c$ individually represent coefficients of the quadratic term, primary term, and constant term, respectively.

**Appendix Table A3.** Coefficients of the power models for NSL data (RCNTL in 2006 was used as the reference image).

| Satellite | Year | $d$ | $e$ | R^2^ |
| --- | --- | --- | --- | --- |
| F10 | 1992 | 1.158 | 1.17 | 0.7955 |
| F10 | 1993 | 2.105 | 0.896 | 0.8103 |
| F10 | 1994 | 1.264 | 1.127 | 0.9093 |
| F12 | 1994 | 3.074 | 0.765 | 0.8909 |
| F12 | 1995 | 1.3 | 1.073 | 0.9151 |
| F12 | 1996 | 1.491 | 1.029 | 0.9018 |
| F12 | 1997 | 0.947 | 1.192 | 0.8952 |
| F12 | 1998 | 0.884 | 1.167 | 0.822 |
| F12 | 1999 | 1.473 | 1.008 | 0.8657 |
| F14 | 1997 | 2.264 | 0.97 | 0.8619 |
| F14 | 1998 | 1.485 | 1.077 | 0.8629 |
| F14 | 1999 | 1.454 | 1.137 | 0.8395 |
| F14 | 2000 | 1.282 | 1.108 | 0.8628 |
| F14 | 2001 | 1.179 | 1.129 | 0.8978 |
| F14 | 2002 | 1.598 | 0.987 | 0.9242 |
| F14 | 2003 | 1.394 | 1.038 | 0.8729 |
| F15 | 2000 | 1.196 | 1.075 | 0.8514 |
| F15 | 2001 | 1.341 | 1.033 | 0.8423 |
| F15 | 2002 | 0.889 | 1.151 | 0.8411 |
| F15 | 2003 | 1.824 | 0.974 | 0.8427 |
| F15 | 2004 | 1.236 | 1.07 | 0.8838 |
| F15 | 2005 | 1.764 | 0.937 | 0.8564 |
| F15 | 2006 | 1.225 | 1.061 | 0.9066 |
| F15 | 2007 | 1.324 | 1.067 | 0.9103 |
| F16 | 2004 | 0.807 | 1.143 | 0.879 |
| F16 | 2005 | 1.058 | 1.119 | 0.9191 |
| F16 | 2006 | 1.005 | 1.117 | 0.9352 |
| F16 | 2007 | 0.965 | 1.109 | 0.8911 |
| F16 | 2008 | 0.855 | 1.108 | 0.948 |
| F16 | 2009 | 0.543 | 1.175 | 0.9176 |
| F18 | 2010 | 0.273 | 1.304 | 0.8914 |
| F18 | 2011 | 0.694 | 1.09 | 0.8351 |
| F18 | 2012 | 0.474 | 1.179 | 0.8743 |
| F18 | 2013 | 0.418 | 1.153 | 0.8501 |

Note: $d$ and $e$ individually represent the constant term and index, respectively.

**Appendix Table A4.** Linear regression results for the national GDP with the sum of DN values in the cross-sectional data.

| Year | Slope | AIC | R^2^ |
| --- | --- | --- | --- |
| 1992 | 0.0021399 (0.00) | 14.54151 | 0.8976 |
| 1993 | 0.0023336 (0.00) | 15.31850333 | 0.8756 |
| 1994 | 0.0027497 (0.00) | 15.88727 | 0.8767 |
| 1995 | 0.0032442 (0.00) | 16.29388333 | 0.8861 |
| 1996 | 0.0036953 (0.00) | 16.57003667 | 0.8912 |
| 1997 | 0.0038611 (0.00) | 16.79868 | 0.8922 |
| 1998 | 0.0040837 (0.00) | 16.95499667 | 0.8924 |
| 1999 | 0.0041885 (0.00) | 17.05823667 | 0.8959 |
| 2000 | 0.0046304 (0.00) | 17.29051667 | 0.896 |
| 2001 | 0.0050124 (0.00) | 17.49525333 | 0.8956 |
| 2002 | 0.0054317 (0.00) | 17.68661333 | 0.8982 |
| 2003 | 0.006137 (0.00) | 17.94116333 | 0.9029 |
| 2004 | 0.0072561 (0.00) | 18.28061333 | 0.9061 |
| 2005 | 0.0084948 (0.00) | 18.61295 | 0.9085 |
| 2006 | 0.0095844 (0.00) | 18.90572667 | 0.9107 |
| 2007 | 0.011017 (0.00) | 19.24521333 | 0.9124 |
| 2008 | 0.0128383 (0.00) | 19.52501 | 0.9171 |
| 2009 | 0.013928 (0.00) | 19.76921 | 0.9114 |
| 2010 | 0.0162368 (0.00) | 20.10145667 | 0.9123 |
| 2011 | 0.0186427 (0.00) | 20.40944 | 0.9143 |
| 2012 | 0.0201241 (0.00) | 20.63242667 | 0.9111 |
| 2013 | 0.0219191 (0.00) | 20.86001 | 0.9076 |
| 2014 | 0.0233322 (0.00) | 21.08367333 | 0.9008 |
| 2015 | 0.0245015 (0.00) | 21.29396 | 0.8915 |
| 2016 | 0.0263207 (0.00) | 21.57368333 | 0.8789 |
| 2017 | 0.0302553 (0.00) | 21.88381 | 0.8769 |

Note: The values in parentheses are p values.

**Appendix Table A5.** Results for the panel regressions.

| Variables | Model (a) | Model (b) | Model (c) | Model (d) |
| --- | --- | --- | --- | --- |
|  | C | C | C | C |
| **Coefficient** | 0.000502*** | 0.000300*** | 0.000300*** | 0.000250*** |
|  | (0.000026) | (0.000016) | (0.000017) | (0.000016) |
| **Constant** | | | | |
| Beijing | -144.5*** |  | -72.70*** | 402.5*** |
|  | (12.06) |  | (14.2) | (117.5) |
| Fujian | -204.9*** |  | -350.0*** | -116.0*** |
|  | (19.39) |  | (41.95) | (33.34) |
| Gansu | -86.99*** |  | -74.80*** | 61.72*** |
|  | (9.896) |  | (7.4) | (17.36) |
| Guangdong | -776.2*** |  | -1133.2*** | -732.8*** |
|  | (59.89) |  | (104.5) | (68.99) |
| Guangxi | -142.2*** |  | -231.4*** | -38.24 |
|  | (14.84) |  | (27.69) | (24.33) |
| Guizhou | 31.38*** |  | -70.42*** | 35.47*** |
|  | (9.92) |  | (16.24) | (13.39) |
| Hainan | -98.12*** |  | -77.98*** | 207.5*** |
|  | (6.349) |  | (6.969) | (47.74) |
| Hebei | -317.2*** |  | -1819.0*** | -1404.8*** |
|  | (52.51) |  | (97.21) | (78.53) |
| Henan | -361.4*** |  | -1008.3*** | -672.4*** |
|  | (41.35) |  | (70) | (62.86) |
| Heilongjiang | -405.1*** |  | -325.7*** | -4.93 |
|  | (31.25) |  | (58.24) | -45.9 |
| Hubei | -77.98*** |  | -472.0*** | -148.9*** |
|  | (20.04) |  | -59.3 | -54.64 |
| Hunan | -38.20** |  | -243.9*** | -67.83** |
|  | (15.79) |  | (45.71) | (28.32) |
| Jilin | -130.8*** |  | -319.7*** | 18.7 |
|  | (16.22) |  | (44.81) | (51.7) |
| Jiangsu | -487.2*** |  | -678.0*** | -490.2*** |
|  | (49.23) |  | (42.98) | (36.88) |
| Jiangxi | -58.35*** |  | -124.4*** | 11.4 |
|  | (10.75) |  | (18.26) | (19.38) |
| Liaoning | -194.8*** |  | -874.2*** | -479.8*** |
|  | (31.43) |  | (61.42) | (58.26) |
| Inner Mongolia | -34.48 |  | -393.6*** | -280.7*** |
|  | (30.37) |  | (22.43) | (18.55) |
| Ningxia | -6.679 |  | -109.3*** | 16.6 |
|  | (7.733) |  | (8.196) | (20.43) |
| Qinghai | -20.92*** |  | -23.57*** | 83.90*** |
|  | (2.681) |  | (2.721) | (16.58) |
| Shandong | -559.6*** |  | -1638.1*** | -1325.9*** |
|  | (66.32) |  | (105.1) | (85.22) |
| Shanxi | -297.7*** |  | -1042.7*** | -629.3*** |
|  | (35.84) |  | (54.45) | (72.04) |
| Shaanxi | -219.9*** |  | -206.6*** | -59.47*** |
|  | (20.07) |  | (18.39) | (17.57) |
| Shanghai | -53.64*** |  | -191.1*** | 178.6** |
|  | (11.49) |  | (13.48) | (72.62) |
| Sichuan | -80.37*** |  | -141.7*** | -3.854 |
|  | (17.13) |  | (34.14) | (21.74) |
| Tianjing | -89.33*** |  | -276.0*** | 75.46 |
|  | (11.09) |  | (20.29) | (51.26) |
| Xinjiang | -242.5*** |  | -254.3*** | -136.0*** |
|  | (22.38) |  | (36.09) | (37.42) |
| Yunan | -187.0*** |  | -127.2*** | 21.73 |
|  | (17.74) |  | (24.52) | (22.69) |
| Zhejiang | -318.7*** |  | -240.5*** | -85.23*** |
|  | (31.26) |  | (27.7) | (19.16) |
| Chongqing | -0.397 |  | -46.98*** | 76.62*** |
|  | (7.177) |  | (13.32) | (19.58) |
| **Variable coefficient** | | | | |
| Beijing |  | -0.000111*** | 0.000046 | -0.00101*** |
|  |  | (0.0000165) | (0.0000368) | (0.000258) |
| Fujian |  | -0.0000801*** | 0.000408*** | 0.0000786 |
|  |  | (0.0000228) | (0.0000603) | (0.0000509) |
| Gansu |  | -0.000019 | 0.000169*** | -0.000230*** |
|  |  | (0.000019) | (0.0000267) | (0.0000574) |
| Guangdong |  | -0.000134*** | 0.000358*** | 0.000219*** |
|  |  | (0.0000187) | (0.000047) | (0.000026) |
| Guangxi |  | -0.0000487** | 0.000366*** | -0.00000107 |
|  |  | (0.0000232) | (0.0000507) | (0.0000519) |
| Guizhou |  | 0.000340*** | 0.000595*** | 0.000108 |
|  |  | (0.0000248) | (0.0000664) | (0.000073) |
| Hainan |  | -0.000197*** | 0.000119*** | -0.00114*** |
|  |  | (0.0000184) | (0.0000316) | (0.000214) |
| Hebei |  | 0.0000161 | 0.00112*** | 0.000898*** |
|  |  | (0.0000269) | (0.0000601) | (0.0000454) |
| Henan |  | -0.0000405* | 0.000649*** | 0.000444*** |
|  |  | (0.0000228) | (0.000051) | (0.0000426) |
| Heilongjiang |  | -0.000136*** | 0.000135*** | -0.000113*** |
|  |  | (0.0000178) | (0.0000495) | (0.0000367) |
| Hubei |  | 0.0000867*** | 0.000827*** | 0.000312*** |
|  |  | (0.000026) | (0.0000997) | (0.0000944) |
| Hunan |  | 0.000136*** | 0.000645*** | 0.000245*** |
|  |  | (0.0000277) | (0.0000905) | (0.0000673) |
| Jilin |  | -0.0000188 | 0.000529*** | -0.0000641 |
|  |  | -0.0000216 | (0.0000803) | (0.0000945) |
| Jiangsu |  | -0.0000472** | 0.000304*** | 0.000236*** |
|  |  | (0.0000213) | (0.0000278) | (0.0000203) |
| Jiangxi |  | 0.0000588** | 0.000383*** | -0.0000296 |
|  |  | (0.0000256) | (0.0000526) | (0.0000685) |
| Liaoning |  | 0.0000265 | 0.000837*** | 0.000488*** |
|  |  | (0.0000234) | (0.0000587) | (0.0000535) |
| Inner Mongolia |  | 0.000193*** | 0.000668*** | 0.000529*** |
|  |  | (0.0000314) | (0.0000361) | (0.0000294) |
| Ningxia |  | 0.000200*** | 0.000784*** | -0.0000682 |
|  |  | (0.0000387) | (0.0000536) | (0.000144) |
| Qinghai |  | 0.0000105 | 0.000228*** | -0.00112*** |
|  |  | (0.000022) | (0.00003) | (0.000221) |
| Shandong |  | -0.0000405* | 0.000686*** | 0.000582*** |
|  |  | (0.0000239) | (0.0000478) | (0.0000359) |
| Shanxi |  | -0.0000308 | 0.000800*** | 0.000492*** |
|  |  | (0.0000229) | (0.0000475) | (0.0000555) |
| Shaanxi |  | -0.0000741*** | 0.000184*** | -0.00000182 |
|  |  | (0.0000207) | (0.000028) | (0.0000259) |
| Shanghai |  | 0.0000798*** | 0.000524*** | -0.000370** |
|  |  | (0.0000185) | (0.000037) | (0.000176) |
| Sichuan |  | 0.0000790*** | 0.000304*** | 0.0000693* |
|  |  | (0.0000222) | (0.0000576) | (0.0000418) |
| Tianjing |  | -0.0000216 | 0.000684*** | -0.000259* |
|  |  | (0.0000232) | (0.0000563) | (0.000143) |
| Xinjiang |  | -0.0000751*** | 0.000216*** | 0.0000818* |
|  |  | (0.0000262) | (0.000046) | (0.0000496) |
| Yunan |  | -0.0000800*** | 0.000109*** | -0.000123*** |
|  |  | (0.0000195) | (0.0000409) | (0.0000391) |
| Zhejiang |  | -0.0000610*** | 0.000135*** | 0.0000258 |
|  |  | (0.0000182) | (0.0000267) | (0.0000165) |
| Chongqing |  | 0.000212*** | 0.000413*** | -0.000247** |
|  |  | (0.0000252) | (0.0000614) | (0.000111) |
| **Year** | | | | |
| 1998 |  |  |  | -9.521* |
|  |  |  |  | (5.399) |
| 1999 |  |  |  | -20.98*** |
|  |  |  |  | (5.349) |
| 2000 |  |  |  | -22.35*** |
|  |  |  |  | (5.946) |
| 2001 |  |  |  | -22.39*** |
|  |  |  |  | (6.522) |
| 2002 |  |  |  | -20.99*** |
|  |  |  |  | (7.608) |
| 2003 |  |  |  | -11.37 |
|  |  |  |  | (8.652) |
| 2004 |  |  |  | -0.0181 |
|  |  |  |  | (8.785) |
| 2005 |  |  |  | 19.80** |
|  |  |  |  | (9.752) |
| 2006 |  |  |  | 26.72** |
|  |  |  |  | (10.69) |
| 2007 |  |  |  | 31.57** |
|  |  |  |  | (12.23) |
| 2008 |  |  |  | 37.72*** |
|  |  |  |  | (12.9) |
| 2009 |  |  |  | 50.70*** |
|  |  |  |  | (13.24) |
| 2010 |  |  |  | 66.87*** |
|  |  |  |  | (14.32) |
| 2011 |  |  |  | 84.15*** |
|  |  |  |  | (15.6) |
| 2012 |  |  |  | 85.85*** |
|  |  |  |  | (15.99) |
| 2013 |  |  |  | 82.45*** |
|  |  |  |  | (16.63) |
| 2014 |  |  |  | 82.60*** |
|  |  |  |  | (17.3) |
| 2015 |  |  |  | 77.20*** |
|  |  |  |  | (17.61) |
| 2016 |  |  |  | 76.41*** |
|  |  |  |  | (18.25) |
| 2017 |  |  |  | 81.98*** |
|  |  |  |  | (18.95) |
| N | 630 | 630 | 630 | 630 |
| R^2^ | 0.953 | 0.929 | 0.992 | 0.996 |
| AIC | 11.11 | 11.53 | 9.41 | 8.88 |

Notes: 1) ***, **, and * denote significance at the 1%, 5%, and 10% levels, respectively. 2) The values in parentheses are standard errors.


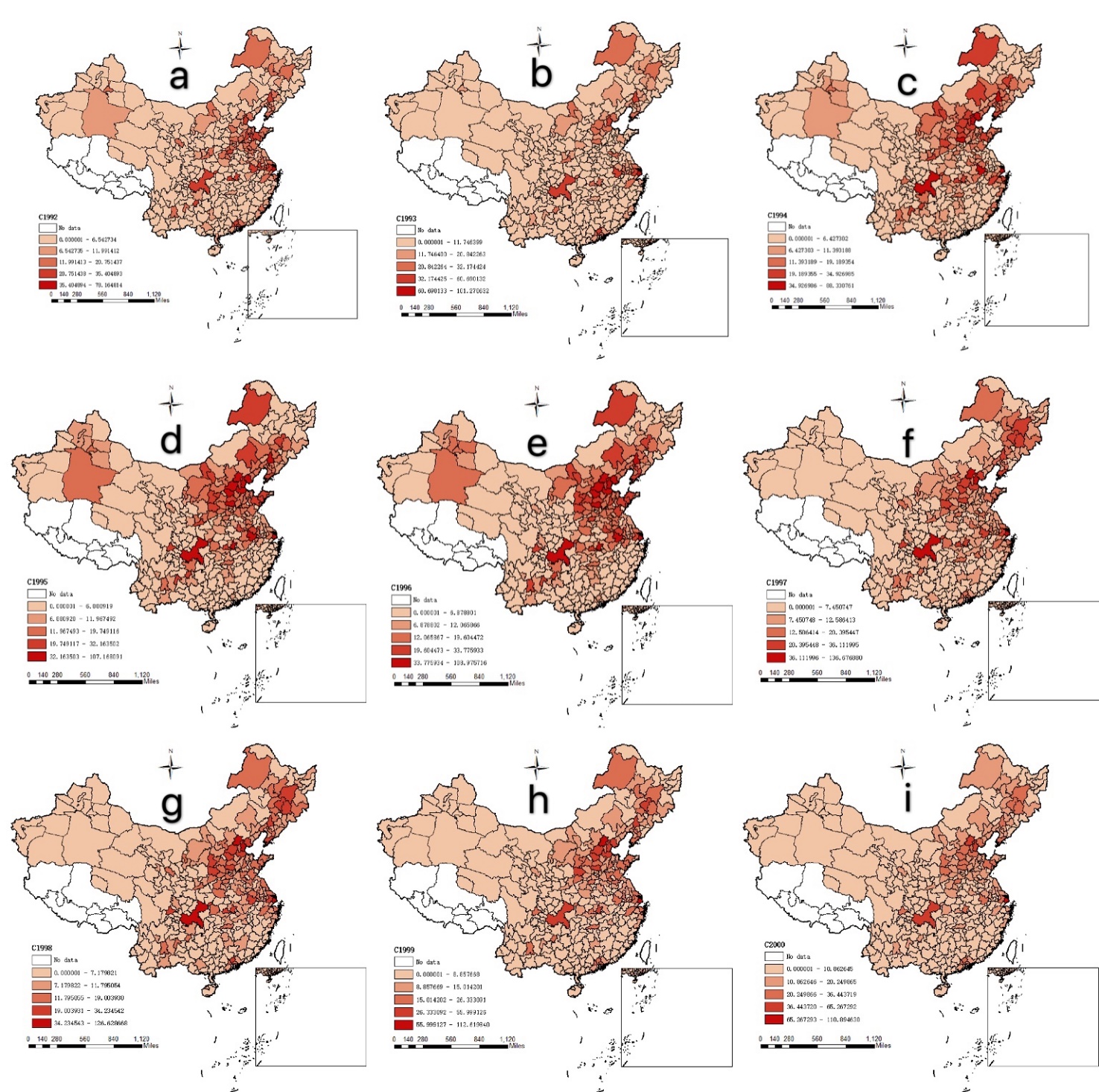


**Appendix Figure 1.** Spatial and temporal pattern of China’s city-level carbon emissions during 1992–2000 (unit: million tons).

Note: These images were made by ArcGIS 10.0. The version is available from: https://www.esri.com/en-us/arcgis/products/arcgis-maps-for-office/download.


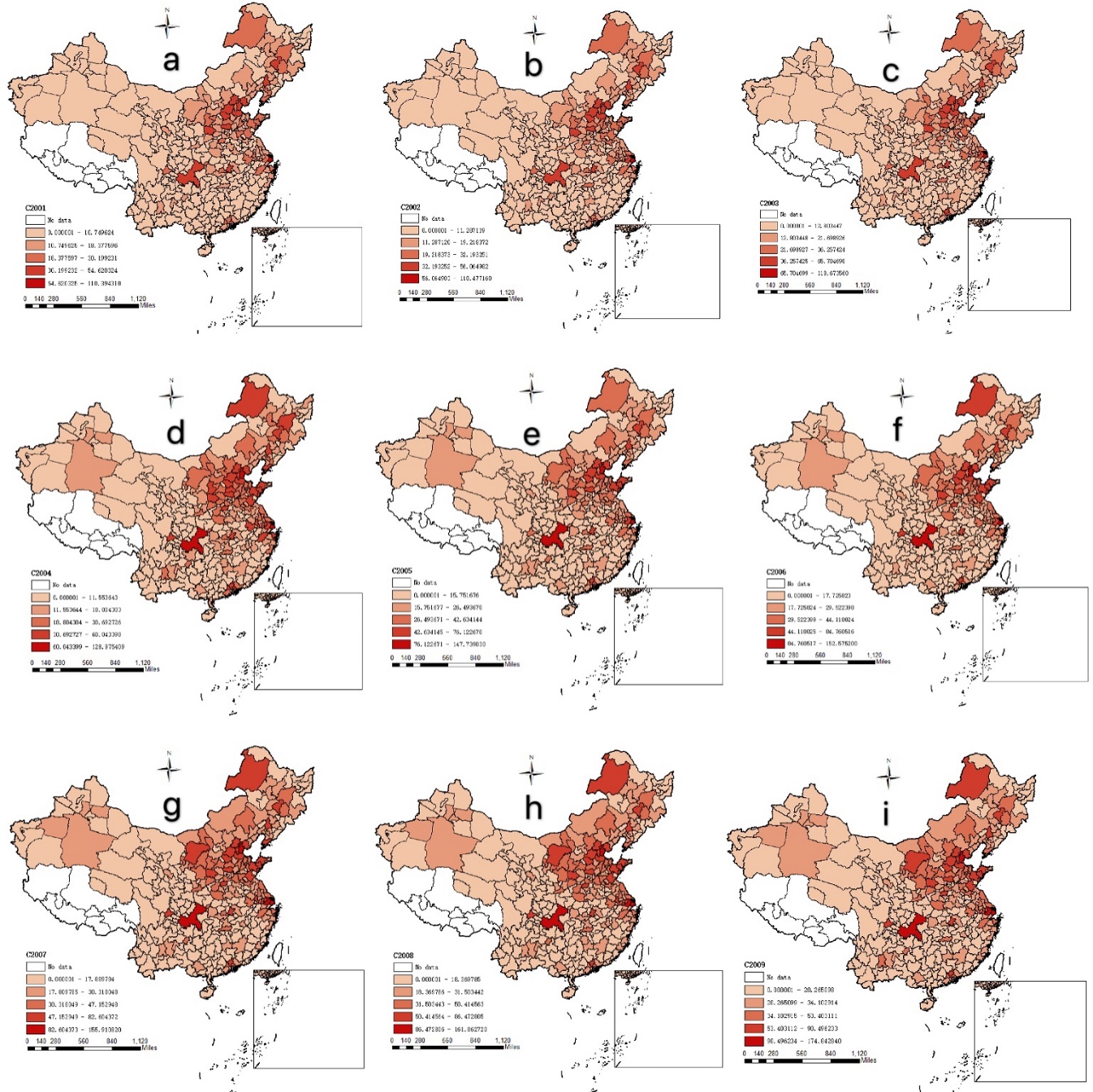


**Appendix Figure 2.** Spatial and temporal pattern of China’s city-level carbon emissions during 2001–2009 (unit: million tons).

Note: These images were made by ArcGIS 10.0. The version is available from: https://www.esri.com/en-us/arcgis/products/arcgis-maps-for-office/download.


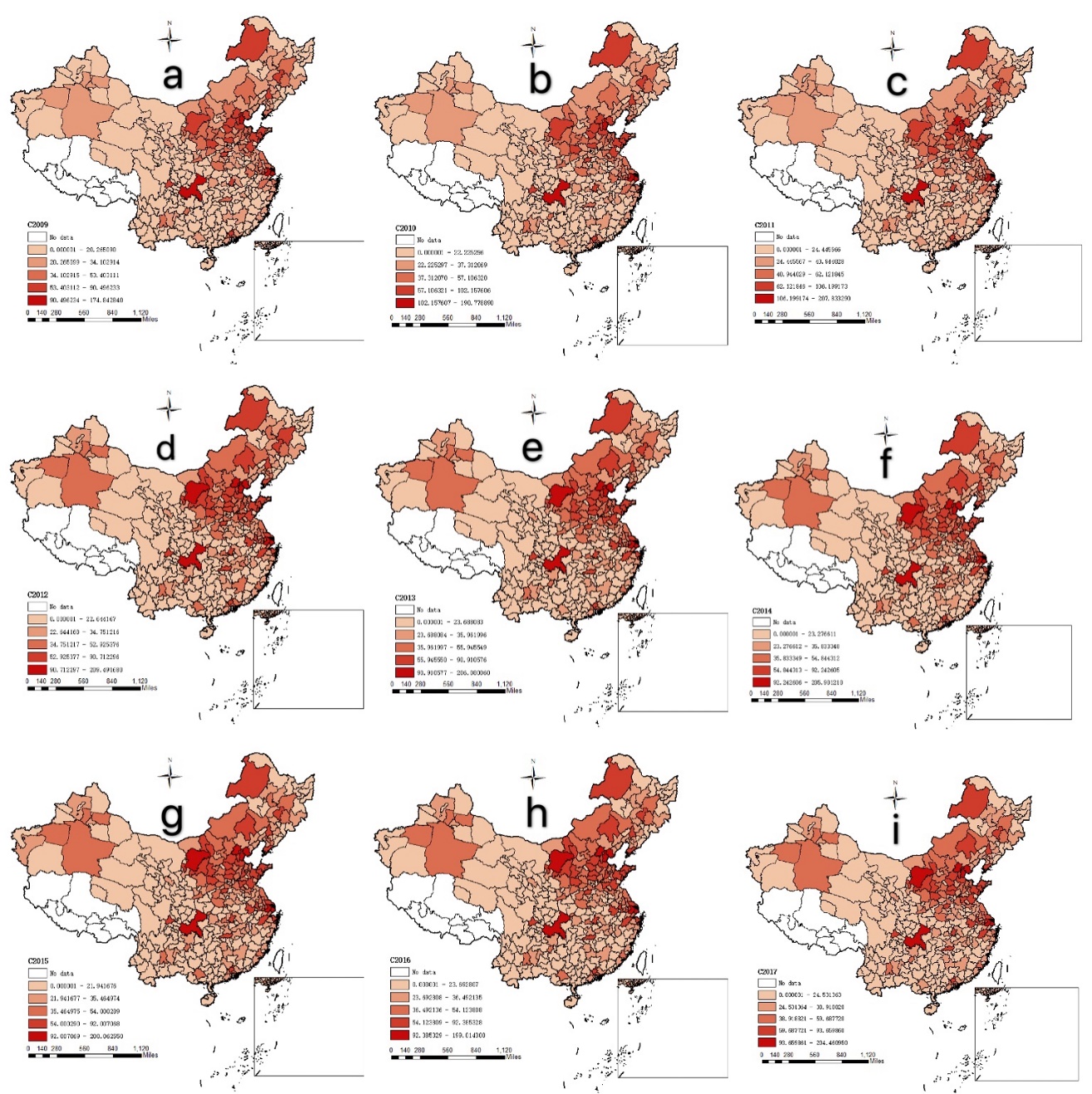


**Appendix Figure 3.** Spatial and temporal pattern of China’s city-level carbon emissions during 2009–2017 (unit: million tons).

Note: These images were made by ArcGIS 10.0. The version is available from: https://www.esri.com/en-us/arcgis/products/arcgis-maps-for-office/download.
